# Disaster risk financing and insurance for earthquake-prone state buildings in Indonesia

**DOI:** 10.4102/jamba.v16i1.1597

**Published:** 2024-07-18

**Authors:** Hesti Marlina, Dina Ruslanjari, Inayah B.A. Hakim

**Affiliations:** 1 Graduate School, Master in Disaster Management Program, Universitas Gadjah Mada, Yogyakarta, Indonesia; 2 Center for Human Resource Trainings, National Search and Rescue Agency of the Republic of Indonesia, Jakarta, Indonesia

**Keywords:** disaster risk financing, earthquake insurance, rapid visual screening, state building, economic policy, state budget

## Abstract

Indonesia situated within the volatile Ring of Fire, faces recurring and devastating earthquakes that pose significant economic threats. The Government of Indonesia (GoI) has initiated a disaster risk financing strategy to address these challenges. However, the implementation of disaster insurance within this strategy remains limited. This study examined the GoI’s disaster risk financing methods, assessed the effectiveness of existing disaster insurance practices, and proposed strategies for improving disaster risk reduction (DRR). Literature review was used to analyse disaster fund and insurance implementation. A building vulnerability simulation, based on Federal Emergency Management Agency’s (FEMA) P-154 rapid visual screening, determined the appropriate financing strategy. State buildings were assessed using nine vulnerability factors and categorised by seismicity. The research found that disaster financing mostly relied on national funds to cover all disaster damages. Existing disaster insurance lacked clarity in defining insurable buildings, resulting in broad inclusion. Vulnerability assessments showed that each building type exhibited different potential earthquake damage levels. Consequently, insurance coverage is recommended for high-damage categories but discouraged for low to moderately vulnerable buildings.

**Contribution:**

This study offered insights into Indonesia’s current disaster risk financing and insurance landscape, and provides a strategic framework for optimising these mechanisms to better protect state buildings from earthquake-related risks.

## Introduction

Indonesia, the world’s largest archipelago, is situated within a highly active seismic zone, characterised by its location amid three major tectonic plates – Eurasia, Indo-Australia and Pacific (Ibrahim & Subardjo 2004). This unique geographical and geological setting results in a notably high frequency of earthquakes, with 5 out of 20 significant global earthquakes reported in proximity to Indonesia (The United States Geological Survey 2019). These seismic events, though a natural occurrence, inflict severe consequences across multiple dimensions, including loss of life, economic setbacks, fiscal stress and delayed developmental progress.

As an illustrative example, the devastating magnitude 9.1 earthquake that struck Aceh and the northern part of Sumatera Island in 2004 led to an estimated loss of approximately 280 000 lives, with nearly 14 000 individuals reported missing, and over one million people displaced from their homes (BMKG 2018). Furthermore, this colossal seismic event left a staggering Indonesian Rupiah (IDR) 22.8 trillion deficit in its wake, significantly straining financial resources (Media Keuangan 2019). The disjunction between economic deficits and available reserve funds prolongs the post-disaster recovery process (BKF 2018). These seismic challenges underscore the critical need for effective disaster risk financing and mitigation strategies tailored to the Indonesian context, for example the protection of state assets through disaster. As emphasised by Kahramanoğlu and Büyüksarıkulak (2019:106–132), a nation’s economic condition is intrinsically influenced by its geographical attributes and available resources. In this context, Gignoux & Menendez (2016) in Mushonga and Mishi (2022) states that the amplification of investments aimed at recovering lost properties and infrastructure paves the way for subsequent economic expansion and a rise in economic engagement.

The assessment of projected building damage because of earthquakes and the economic losses resulting from such damage serve as a reference point for determining financing strategies in this study. Organisation for Economic Co-operation and Development (OECD 2018) stated that a comprehensive assessment of earthquake risk, encompassing building vulnerability, potential economic losses, is a prerequisite for formulating disaster financing strategies. Information derived from this assessment can be used to make decisions regarding various financing options, including the management of contingency funds or the transfer of earthquake risk through insurance.

In Indonesia, the disaster risk financing framework comprises five distinct layers, encompassing state and local budgets, pooling funds, contingency loans, insurance and international relief (BKF 2018), with the state budget serving as the primary mechanism. The implementation of disaster risk financing for state assets has been initiated progressively, commencing in 2020, with initial efforts focussed on safeguarding Ministry of Finance buildings (Inarko 2018; Kemenkeu 2019). This phased approach underscores the country’s commitment to fortify its financial resilience against the potentially devastating impact of natural disasters on its critical infrastructure. At regional level, Haris et al. (2023) found out that enhancing disaster resilience and prioritising risk management should be given greater consideration when allocating funds for regional disaster management. However, Coetzee et al. (2023:398–412) investigated that the budget allocation for disaster risk reduction (DRR) has mostly been allocated for response operations, with a smaller proportion for pre-disaster practices.

Government of Indonesia should incorporate medium- to long-term financing schemes into their disaster-prevention planning strategies to mitigate the adverse fiscal impacts of disasters while also considering the adoption of alternative financing instruments like disaster insurance and bond issuance (Wiyanti & Halimatussadiah 2021). Disaster financing in Indonesia draws from diverse sources, including state funds, local budgets and contributions from the community. Community funds are gathered from private individuals, business entities, non-profit organisations, both at the local and international levels (Kemenkeu 2019). These funds manifest in three distinct forms: contingency funds allocated for preparedness before a disaster strikes, on-call funds for emergency response and social assistance in the form of grants for post-disaster relief. Notably, disaster assistance in Indonesia is predominantly supported by the national budget (Inarko 2018). This support extends to covering the repair of homes owned by members of the community, as well as providing guarantees for state and local government buildings.

**FIGURE 1:**
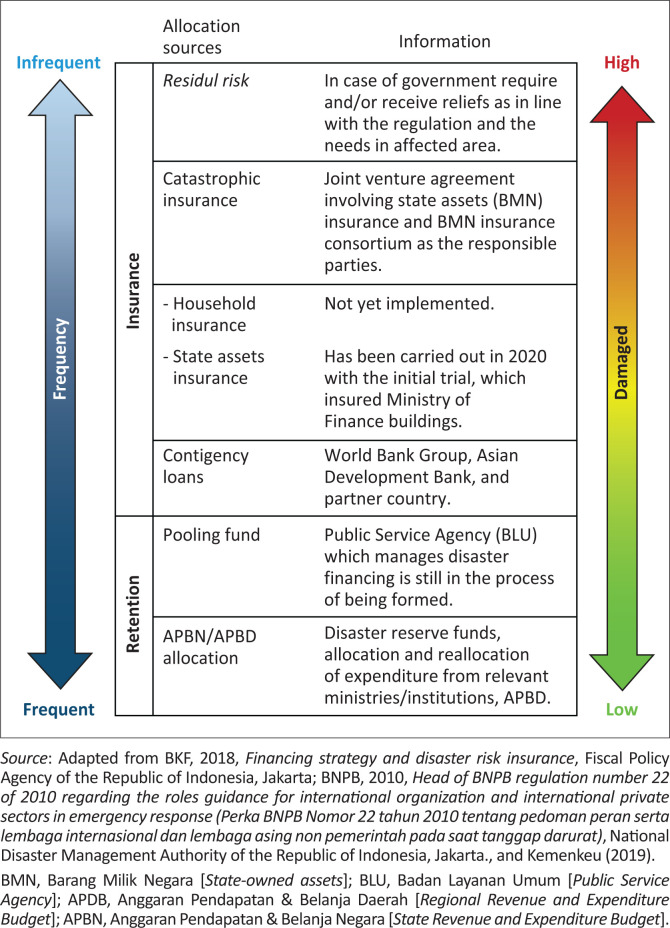
Disaster funding sources layering.

According to the World Bank and Asian Development Bank (ADB 2017), the optimal approach to disaster risk financing involves a collaborative framework that combines retention expenditure with insurance mechanisms. Government of Indonesia has categorised the sources of disaster financing based on a risk layering concept, as illustrated in Figure 1. For government-owned damaged buildings, the emphasis remains on retention instruments, which entail budget reallocation. Ministries and institutions propose budget allocations for building reconstruction to the Directorate General of Budget within the Ministry of Finance Indonesia. This approach underscores the importance of a coordinated strategy in managing disaster-related financial challenges effectively.

The layering of disaster funding sources, as detailed in Figure 1, underscores the importance of budget planning. This approach yields several significant benefits, including the augmentation of disaster reserve fund allocations, a reduction in risk exposure and the fostering of disaster mitigation endeavours. Furthermore, it is imperative that such budget planning is underpinned by regulations that are both acceptable and implementable, garnering support from both the public and private sectors.

The synergy between effective budget planning and policies supported by both public and private stakeholders plays a pivotal role in fostering long-term fiscal stability (Phaup & Kirschner 2010:1–24). Regrettably, the government has yet to fully implement this layering approach. Currently, practical disaster funding sources such as state and local budget allocations, contingency loans and residual risk are underutilised. The government’s reliance on the state budget to address all frequencies and impacts of disasters has consequently led to a pronounced financial gap.

The government has devised a pooling fund to oversee disaster funds, as outlined in BKF (2018). Insurance financing has been gradually introduced by the government since 2020, initially for the Ministry of Finance buildings, with plans to extend state asset insurance coverage to all government buildings nationwide (Kemenkeu 2019).

Enhancing disaster risk financing in Indonesia relies on the efficacy of government strategies. This necessitates a focus on financial governance, fostering innovation, forming partnerships with the private sector and leveraging technological advancements (Roshanaei & Khoramshahi 2020:108–203). Additionally, the availability of information technology and accessible post-disaster data is paramount in facilitating the successful execution of disaster recovery initiatives (Omar, Alijani & Mason 2011:127–141).

## Research methods and design

### Material and methods: Federal Emergency Management Agency’s P-154 rapid visual screening

The study conducts an extensive literature review, scrutinising disaster layering financing concepts that are relevant and implementable in Indonesia and other comparable regions. Furthermore, it delves into the analysis of building vulnerability using FEMA’s P-154 Rapid Visual Screening (RVS), exploring various plausible scenarios and accounting for building vulnerability alongside seismicity levels. The assessment is based on the observation of building components without involving structural calculations. The inspection is conducted by identifying building components recorded in a form developed by FEMA. Ultimately, the study aims to provide valuable insights and recommendations regarding the most suitable and pertinent disaster risk financing strategy, tailored to the specific needs and conditions of earthquake-prone areas in Indonesia.

Similarly, these methods are employed to examine the landscape of disaster insurance in Indonesia, particularly concerning earthquake-induced damage to state-owned buildings. While regulations pertaining to insurance issues in Indonesia have been progressively introduced, the specific framework for state-owned buildings was established in 2020. This discussion will delve into the challenges posed by existing general earthquake insurance and the unique considerations relevant to state-owned structures.

In the FEMA P-154 RVS analysis, state buildings are simulated across five seismic layers: low, moderate, moderately high, high and very high. Additionally, building vulnerability factors, including building characteristics, height, structural susceptibility, adherence to pre-code standards, post-benchmark performance and soil condition, are examined across nine common building conditions found in Indonesia (refer to Table 1). The parameters examined by FEMA P-154 include both architectural and structural factors that can be visually observed. Federal Emergency Management Agency P-154 has meticulously defined the values of each parameter that influences building performance during an earthquake. These parameters include the following: seismic hazard level, building type, soil type, building height, spacing between buildings, vertical irregularity, plan irregularity, threat of falling from outside the building, guidelines or standards used during construction and final score.

**TABLE 1:** Building vulnerability factor.

Building	Type	Number of floor(s)	Complexity		Pre-code	Post benchmark	Soil type
			Horizontal	Vertical			
Building A	C1	1 floor	-	-	yes	-	Type D
Building B	C1	1 floor	-	-	-	yes	Type D
Building C	C1	3 floors	-	- Short column	-	yes	Type E
Building D	C1	3 floors	Irregular floor plan	- Soft story	-	yes	Type D
Building E	S1	10 floors	-	-	-	yes	Type E
Building F	S1	10 floors	-	- Out plan set back	-	yes	Type D
Building G	S2	10 floors	-	- Soft story	-	yes	Type D
Building H	S2	10 floors	Irregular floor plan	- Split level - Out plan set back	-	yes	Type D
Building I	S2	10 floors	Irregular floor plan	- Split level - Soft story	-	yes	Type E
-	C1	-	Concrete moment-resisting frames	-	-	-	Type D: Stiff soil
-	S1	-	Braced steel frame	-	-	-	Type E: Soft soil
-	S2	-	Steel moment-resisting frame	-	-	-	-

Some advantages of RVS include the following: Minimal equipment is required. The screening process is non-destructive. It requires minimal human resources. The implementation time is quick, and it has a low level of disruption to activities. Eren and Lus (2014) employed the method to determine the percentage of maximum loss or probability maximum loss (PML) resulting from earthquake-induced damage to industrial buildings. The analysis concluded that forecasting maximum losses is crucial for estimating the economic loss incurred from such building damage. Additionally, the forecasted maximum losses are also utilised in calculating the insurance premiums to be paid. This study involves a simulation of the vulnerability of buildings under different seismic conditions presented in Indonesia. The inspection team conducting RVS consists of individuals who are knowledgeable in the application of FEMA P-154 RVS and possess civil engineering expertise.

Siddiq (2006) in his research found that the building type that experienced significant damage during the 2006 Yogyakarta Earthquake was the residential structure predominantly employing brick as the structural wall material (bearing vertical loads from the roof or multi-story floor + roof), timber frame + boards or thatch, timber frame + half-brick structural wall or reinforced concrete frame + half-brick structural wall (with a wall thickness of 12 cm–15 cm), categorised as building type C1 or concrete frame structures. On the other hand, structures with types S1 and S2, or earthquake-resistant concrete frames or shear walls, were identified in larger and/or multi-story buildings (2–6 stories or Engineered Structures, ES).

Hakim (2019) stated in his book that the predominant soil types in Indonesia are Inceptisols covering 38.51% of the total area and Ultisols covering 24.27%. Inceptisols tend to be softer compared to some older and more developed soil orders, and generally, Inceptisols can be categorised as relatively soft soils compared to other soil orders. This soil type falls into Soil Type E or soft soil category. Meanwhile, Ultisols have a high clay mineral content, are acidic in nature and generally can be considered as hard soils falling into Soil Type D or stiff soil category.

Building characteristics are categorised as type C1, S1 and S2, where C1 represents moment-resisting concrete frame structures, S1 denotes moment-resisting steel frame structures, and S2 indicates reinforced steel frame structures. These three types are officially approved for state-owned buildings (PUPR 2018). Soil conditions in this study are assumed to be Type D, characterised as dense soil, and Type E, recognised as soft soil derived from sedimentary materials such as loose sand and river sediment, often found above Type D. This type of soil condition is suitable for one- and two-story buildings with a height of less than 25 m above ground level. However, it does not directly contribute to the final vulnerability score. Type E soil condition is primarily examined to ascertain its impact on building vulnerability (FEMA 2015). The specific building vulnerability factors are detailed in Table 1.

The ultimate outcome derived from FEMA’s P-165 RVS assessment is then categorised into five distinct classes to discern varying levels of building damage potential. The classification of damage potentials in this study aligns with the framework proposed by Nanda and Majhi (2014: 2218–2226), which is comprehensively outlined in Table 2. It is important to note that the damage possibilities determined in this study do not directly equate to actual damages, as the RVS procedure focusses solely on building vulnerability factors. Consequently, the combined results of FEMA’s P-154 and the building vulnerability estimation serve as a valuable tool for formulating a disaster financing strategy, providing actionable insights to guide government decision-making in this regard.

**TABLE 2:** Rapid visual screening score and damage potentials.

RVS score	Damage potential
S > 2.5	Slight damage
2.0 < S < 2.5	Moderate damage
0.7 < S < 2.0	Heavy damage
0.3 < S < 0.7	Very heavy damage
S < 0.3	Collapse

*Source*: Nanda, R.P. & Majhi, D.R., 2014, ‘Rapid seismic vulnerability assessment of building stocks for developing countries’, *KSCE Journal of Civil Engineering* 18, 2218–2226. https://doi.org/10.1007/s12205-014-0050-0

### Ethical considerations

This article followed all ethical standards for research without direct contact with human or animal subjects during the field research.

## Results

A comprehensive assessment of building and structural vulnerability, along with an evaluation of potential economic losses, stands as an essential prerequisite for the development of effective disaster financing strategies and informed financial decision-making (OECD 2018). To assess the vulnerability of state-owned buildings, this study employs the RVS procedure outlined in FEMA P-154 working papers. This inspection method involves the observation of building components while excluding structural calculations, rendering it suitable for initial building assessments (FEMA 2020).

In this research, state-owned buildings are categorised into nine distinct conditions. Moreover, the vulnerability of each state-owned building is calculated across five seismic conditions, encompassing (1) low seismicity, (2) moderate seismicity, (3) moderately high seismicity, (4) high seismicity and (5) very high seismicity (see Table 3).

**TABLE 3:** Federal emergency management agency P-154 rapid visual screening calculation.

Building	Level of Seismicity									
	Low seismicity		Moderate seismicity		Moderate high seismicity		High seismicity		Very high seismicity	
	SL1	*p*	SL1	*p*	SL1	*p*	SL1	*p*	SL1	*p*
Building A	NA	Slight	1.3	Heavy	1.3	Heavy	1.1	Heavy	0.9	Heavy
Building B	5.6	Slight	4.1	Slight	3.6	Slight	3.4	Slight	2.4	Moderate
Building C	3.0	Slight	2.3	Moderate	2.4	Moderate	2.5	Slight	1.6	Heavy
Building D	3.3	Slight	2.2	Moderate	1.7	Heavy	1.9	Heavy	1.1	Heavy
Building E	4.5	Slight	3.3	Slight	3.1	Slight	3.5	Slight	2.2	Moderate
Building F	4.4	Slight	3.0	Slight	2.6	Slight	2.5	Slight	1.7	Heavy
Building G	4.2	Slight	2.9	Slight	2.6	Slight	1.8	Heavy	1.8	Heavy
Building H	2.3	Moderate	1.3	Heavy	1.2	Heavy	1.0	Heavy	0.9	Very Heavy
Building I	0.9	Heavy	0.4	Very Heavy	0.7	Very Heavy	0.4	Very Heavy	0.6	Very Heavy

S_*L1*_, Final score level 1; P, damage potential.

### Low seismicity

Buildings situated in areas with low seismic activity typically exhibit an acceleration spectral value of S_s_ less than 0.250 g and S_1_ less than 0.100 g. In accordance with FEMA (2015) guidelines, construction in such regions typically does not necessitate stringent seismic design requirements. The RVS assessment results for Buildings A, B, C, D, E, F, G and H reveal final scores exceeding 2, indicating that these eight buildings are unlikely to collapse during an earthquake. These structures are expected to incur minor damage, with the exception of Building H, which is projected to experience moderate damage. Conversely, Building I obtains a vulnerability score of less than 2, suggesting a potential for collapse and the likelihood of significant damage in the event of an earthquake.

The overall RVS scores affirm the structural integrity of the nine building conditions assessed. Building A, despite being constructed prior to the implementation of modern building codes, remains deemed safe for use in low seismicity zones. A similar outcome applies to Building H, which exhibits severe and moderate horizontal and vertical irregularities but remains structurally sound. It is essential to note that the scenario may differ when considering buildings in regions with higher seismic activity.

### Moderate seismicity

The outcomes of the RVS calculations reveal that six buildings obtained final scores exceeding 2 (*S*_*L1*_ > 2). Among these, four buildings, namely Buildings B, E, F and G, are anticipated to experience minor damage, while Buildings C and D are projected to incur moderate damage. These six structures are classified as resistant to collapsing in the event of an earthquake. Conversely, Buildings A, H and I received final scores below 2 (*S*_*L1*_ < 2), indicating their susceptibility to collapse during an earthquake event. Buildings A and H are at risk of suffering significant damage, while Building I exhibits the potential for very heavy damage. Consequently, the structural integrity of Buildings A, H and I does not warrant their utilisation in regions characterised by moderate to very high seismicity.

### Moderate high seismicity

This level reveals that among the examined buildings, five of them attained a final score exceeding 2, whereas the remaining structures received scores below 2. Specifically, Building I is at significant risk of experiencing substantial damage, while Buildings A, D and H are anticipated to incur severe structural damage. Consequently, it is advisable to refrain from employing the structural designs used in these four buildings within regions characterised by moderately high seismic activity. Conversely, the structural designs of Buildings C, E, F and G appear to meet acceptable criteria for use in areas with moderately high seismicity.

### High seismicity

Buildings situated in high seismic zones typically incur substantial damage, with a subset of structures experiencing exceptionally severe damage. Nevertheless, there exist four building scenarios where the potential for minor damage exists because of the limited influence of the vulnerability factor on building collapse. Conversely, buildings characterised by the presence of multiple severe vulnerability factors substantially elevate the risk of structural collapse. For instance, consider Building I, which exhibits both moderate and severe horizontal and vertical irregularities and is constructed on Type E soil or soft ground. This particular configuration renders Building I highly susceptible to severe damage in the event of an earthquake. Eren and Lus (2014) revealed that industrial buildings with a high vulnerability probability are projected to require larger repair costs. This implies that the economic losses because of building damage are substantial, necessitating significant repair expenses.

### Very high seismicity

Buildings located in regions characterised by very high seismic activity have the potential to experience varying degrees of damage, which encompass moderate, heavy and very heavy damage. Buildings categorised as having the potential for heavy damage typically exhibit both vertical and plan irregularities. This situation becomes more critical if the building is constructed on Type E soil or soft ground. The use of government-owned asset insurance rates is appropriately applied to areas with very high hazard levels. Dorojatun and Kurniawan (2014) discovered in their research that buildings with a high vulnerability probability incur greater economic losses compared to those with low vulnerability probabilities.

### Final score of buildings vulnerability

**FIGURE 2:**
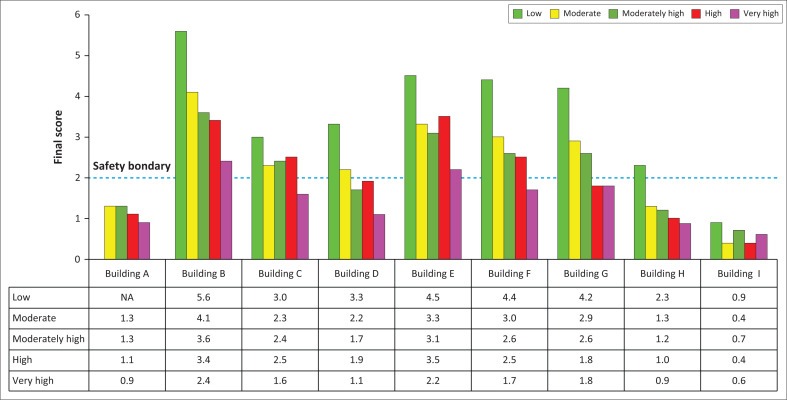
Final score of buildings vulnerability based on seismicity levels.

The final score serves as an indicator, revealing that as the seismic activity level increases, the likelihood of the building collapsing during an earthquake also rises. Furthermore, the vulnerability factor of the building exerts a considerable influence on both the final score and the potential damage it faces. Figure 2 visually presents the final building scores corresponding to each seismic level.

Buildings designated as Building B and Building E exhibit notable resilience, with their vulnerability factors indicating that they are unlikely to undergo structural collapse in the event of an earthquake. These two buildings possess structural characteristics and vulnerability profiles suitable for operation across various seismic levels. Notably, they lack horizontal and vertical irregularities and have been constructed in accordance with earthquake-resistant building standards, with foundations on stable, hard ground.

In contrast, Building I demonstrates a higher vulnerability factor, primarily because of the presence of both moderate and severe vertical irregularities, irregular floor plans, and its construction on soft soil deposits adjacent to riverbeds. Conversely, Building A, despite the absence of horizontal and vertical irregularities and its foundation on solid ground, receives a final score of less than 2. This discrepancy can be attributed to the building’s vulnerability arising from non-compliance with earthquake-resistant building standards during its construction.

The vulnerability score, as illustrated in Figure 2, reveals an inverse relationship between seismic intensity and the score, signifying an increased risk of building collapse as seismic activity rises. This risk is further compounded when buildings exhibit vulnerability factors, including building type, horizontal and vertical irregularities, adherence to earthquake-resistant building standards and soil type.

When formulating an effective disaster financing strategy, it becomes imperative to account for potential building damage, allowing governments to estimate the necessary financial preparations in case of an actual disaster. This consideration extends to insurance, where anticipatory assessment of potential costs aids in ensuring that premiums do not surpass the benefits that can be received. Marlina (2020) found that using insurance premium rates for areas with low seismic hazard or low to moderate hazard levels, as well as moderate to high hazard levels, is less effective.

### Building damage prediction

The findings indicate a correlation between higher seismic levels and increased damage potential. Nevertheless, the degree of potential damage varies among buildings, even when located within areas sharing the same level of seismicity. This assertion is substantiated in Table 4.

**TABLE 4:** Building damage prediction.

Building	Seismicity level				
	Low	Moderate	Moderately high	High	Very high
Building A	Slight	Heavy	Heavy	Heavy	Heavy
Building B	Slight	Slight	Slight	Moderate	Moderate
Building C	Slight	Moderate	Moderate	Heavy	Heavy
Building D	Slight	Moderate	Heavy	Heavy	Heavy
Building E	Slight	Slight	Slight	Moderate	Moderate
Building F	Slight	Slight	Slight	Heavy	Heavy
Building G	Slight	Slight	Slight	Heavy	Heavy
Building H	Moderate	Heavy	Heavy	Very heavy	Very heavy
Building I	Heavy	Very heavy	Very heavy	Very heavy	Very heavy

Green = slight; yellow = moderate; orange = heavy; red = very heavy.

Table 4 provides insights into the expected damage levels of buildings situated in regions characterised by varying degrees of seismic activity. Different levels of damage may occur to both structural and non-structural components. A slight level of damage indicates that structural elements remain intact, with only minor harm to non-structural elements, such as a few cracks on the walls and a small piece of plaster detaching. Moderate level refers to structural components experience minor impairment, while non-structural components are expected to suffer moderate damage, including numerous wall cracks, the dislodging of sizable plaster sections and the potential collapse of non-structural elements like cladding and parapets.

In cases of heavy damage, there is a range from partial to severe harm, with substantial cracks spanning most walls, roof collapses dislodging from their supports and fractures affecting cladding and parapet components, accompanied by the failure of non-structural elements like partitions and canopies. The most severe level, very heavy damage, entails significant structural deterioration and extensive harm to non-structural components, exemplified by severe wall damage characterised by extensive cracks and partial structural failures affecting roofs and floors.

Buildings B and E demonstrate a high level of safety across three tiers of seismicity. These structures exhibit no discernible vertical or horizontal irregularities, which typically contribute to vulnerability. Conversely, Building I, despite being situated in an area with low seismic activity and adhering to earthquake-resistant building standards, ranks as the least secure. This lower level of safety arises from additional factors such as horizontal and vertical irregularities and the presence of soft ground types in its vicinity.

Concerning disaster funding strategies, insurance plays a pivotal role in financing high-impact disaster risks, whereas smaller-scale incidents rely on state budget allocations. Consequently, not all state buildings warrant insurance coverage. Instead, it is advisable to insure buildings categorised as having the potential for heavy or very heavy damage, while those projected to sustain minor or moderate damage should be excluded from coverage.

The categorisation of damage as either slight or moderate aligns with the appropriateness of financing options, with risk financing from the state budget being more suitable in such cases. This approach effectively minimises state budget expenditures, as these funds are only disbursed when a disaster event occurs. This differs from disaster risk financing through insurance, which necessitates annual premium payments regardless of whether a risk materialises.

Dorojatun and Kurniawan (2014:1–7) assert that the insurance coverage for state buildings should be tailored to the vulnerability assessment of each individual property. In essence, buildings with a low level of vulnerability may not benefit significantly from insurance coverage. Consequently, the key determinants for disaster financing include both the assessment of building vulnerability and the level of seismic activity.

One potential mechanism for estimating potential building damage is FEMA’s P-154 RVS, as demonstrated by Eren and Lus (2014) in their study on industrial buildings in Turkey. Their findings reveal that each applicable approach yields distinct levels of building vulnerability. Consequently, the research places significant emphasis on assessing building risk to precisely specify insurance protection and determine the appropriate premium rate.

In conclusion, this research suggests that providing insurance coverage for all state buildings in Indonesia may not be the most effective approach, as only select properties are situated in high seismicity zones. Nevertheless, the GoI has the option to issue an ‘all-risk’ regulation if it intends to extend insurance coverage to all state buildings, regardless of the types of disaster risks involved. In such a scenario, the premium rate should align with the associated risk levels.

## Conclusion

The GoI has established a layered mechanism for disaster financing to bridge the fiscal gap. Nonetheless, disaster funding still heavily relies on the state budget to address various disaster frequencies and impacts. As part of this new financing approach, insurance has been introduced for the Ministry of Finance building, offering significant potential benefits for mitigating disaster risks in Indonesia. Insurance financing is expected to expedite post-disaster recovery and reduce dependence on the national budget. However, insuring all state buildings in the country presents additional challenges, particularly in terms of budgeting for insurance premiums. The specific criteria and priorities for selecting buildings to be insured are not clearly defined. Furthermore, insurance rates are uniformly applied across all regions and levels of disaster risk. In practice, the results of building vulnerability assessments using FEMA’s RVS P-154 method demonstrate that potential damage varies for buildings within the same seismic area, even though they share similar geographical characteristics. Buildings classified as having severe and very severe damage potential typically exhibit vulnerability factors such as vertical and horizontal irregularities, construction on soft ground, non-compliance with earthquake-resistant building standards and variations in building types. Therefore, the decision to insure state buildings should not solely be based on the level of disaster threat; the physical condition of the building must also be carefully considered. The research findings suggest that buildings with slight and moderate potential damage should not be insured, while those with the potential for heavy and very heavy damage are recommended for insurance coverage.

A limitation of this study lies in the assessment of building vulnerability, which was conducted through a RVS using parameters from FEMA P-154 RVS. To obtain a vulnerability estimate closer to the actual conditions, the author suggests employing a method capable of examining the structural components of the building in more detail.
